# 1-[(Pyridin-3-yl)(pyrrolidin-1-yl)meth­yl]naphthalen-2-ol

**DOI:** 10.1107/S1600536812033363

**Published:** 2012-08-01

**Authors:** Qin-Qin Zhou

**Affiliations:** a Ordered Matter Science Research Center, College of Chemistry and Chemical, Engineering, Southeast University, Nanjing 211189, People’s Republic of China

## Abstract

The title compound, C_20_H_20_N_2_O, was synthesized by a solvent-free one-pot three-component domino reaction of naph­tha­len-2-ol, nicotinaldehyde and pyrrolidine. The dihedral angle between the naphthalene ring system and the pyridine ring is 74.22 (6)°. The pyrrolidine ring assumes an envelope conformation with the N atom as the flap. An intra­molecular O—H⋯N hydrogen bond stabilizes the mol­ecular conformation.

## Related literature

For the synthesis and structure of a related compound, see: Wang (2012 ▶).
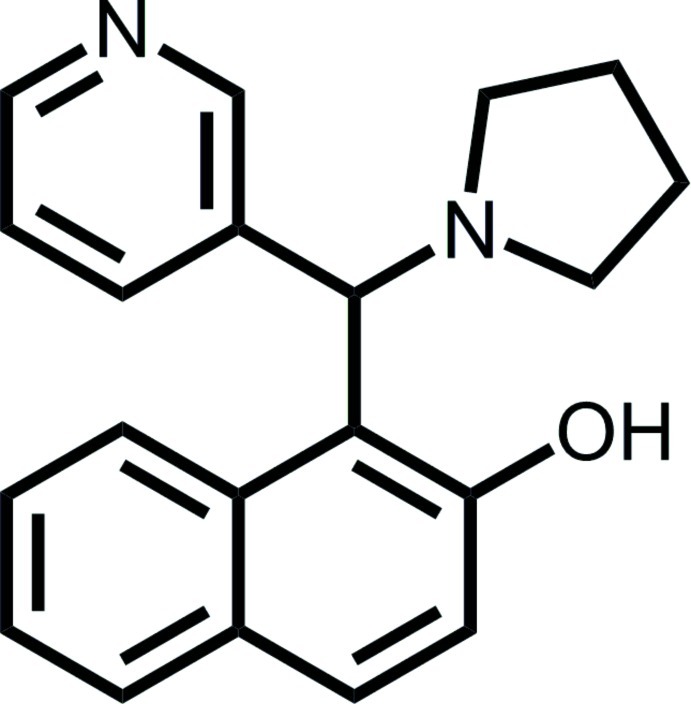


## Experimental

### 

#### Crystal data

C_20_H_20_N_2_O
*M* *_r_* = 304.38
Monoclinic, 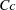

*a* = 9.966 (2) Å
*b* = 15.587 (3) Å
*c* = 10.477 (2) Å
β = 91.60 (3)°
*V* = 1626.9 (6) Å^3^
*Z* = 4
Mo *K*α radiation
μ = 0.08 mm^−1^
*T* = 293 K
0.38 × 0.32 × 0.27 mm

#### Data collection

Rigaku SCXmini CCD diffractometer
Absorption correction: multi-scan (*CrystalClear*; Rigaku, 2005 ▶) *T*_min_ = 0.967, *T*_max_ = 0.982
8194 measured reflections
1857 independent reflections
1361 reflections with *I* > 2σ(*I*)
*R*_int_ = 0.067

#### Refinement

*R*[*F* ^2^ > 2σ(*F* ^2^)] = 0.050
*wR*(*F* ^2^) = 0.122
*S* = 1.02
1857 reflections
209 parameters
2 restraints
H-atom parameters constrained
Δρ_max_ = 0.13 e Å^−3^
Δρ_min_ = −0.13 e Å^−3^

### 

Data collection: *CrystalClear* (Rigaku,2005 ▶); cell refinement: *CrystalClear*; data reduction: *CrystalClear*; program(s) used to solve structure: *SHELXS97* (Sheldrick, 2008 ▶); program(s) used to refine structure: *SHELXL97* (Sheldrick, 2008 ▶); molecular graphics: *DIAMOND* (Brandenburg & Putz, 2005 ▶); software used to prepare material for publication: *SHELXL97*.

## Supplementary Material

Crystal structure: contains datablock(s) I, global. DOI: 10.1107/S1600536812033363/mw2063sup1.cif

Structure factors: contains datablock(s) I. DOI: 10.1107/S1600536812033363/mw2063Isup2.hkl

Supplementary material file. DOI: 10.1107/S1600536812033363/mw2063Isup3.cml

Additional supplementary materials:  crystallographic information; 3D view; checkCIF report

## Figures and Tables

**Table 1:** Hydrogen-bond geometry (Å, °)

*D*—H⋯*A*	*D*—H	H⋯*A*	*D*⋯*A*	*D*—H⋯*A*
O1—H1⋯N1	0.82	1.85	2.572 (3)	147

**Fig. 1.**
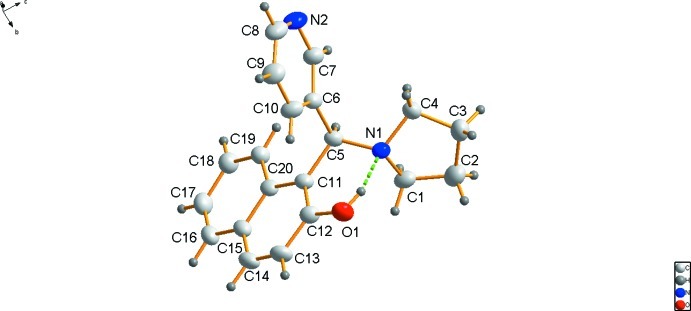
The structure of the title compound with displacement ellipsoids drawn at the 30% probability level. The intramolecular hydrogen bond is shown as a dashed line.

## supplementary crystallographic information

### Comment

The so-called Betti base derivatives, which can be synthesized by many routes (Wang, 2012), have been of great interest in coordination chemistry. Herein the crystal structure of one such compound, obtained by a solvent-free, one-pot, three-component, domino reaction of naphthalen-2-ol, nicotinaldehyde and pyrrolidine is reported.

In the title compound the bond lengths and angles are well within the expected ranges. The dihedral angle between the naphthalene ring system and the pyridine ring is 74.22 (6)°. The pyrrolidine ring adopts an envelope conformation. An intramolecular O—H···N hydrogen bond (Table 1) stabilizes the molecular conformation.

### Experimental

A dry 50 mL flask was charged with nicotinaldehyde (10 mmol), naphthalen-2-ol (10 mmol) and pyrrolidine (10 mmol). The mixture was stirred at 100°C for 5 h and then ethanol (15 mL) was added. After refluxing for 30 minutes, the solution was filtered and crystals of the title compound suitable for X-ray analysis were obtained by slow evaporation.

### Refinement

All H atoms were calculated geometrically and refined using a riding model with C—H = 0.93–0.98 Å, O—H = 0.82 Å and with *U*_iso_(H) = 1.2 *U*_eq_(C)for carbon-bound or 1.5 *U*_eq_ (O) for oxygen-bound H atoms.

### Crystal data

**Table:**

C_20_H_20_N_2_O	*F*(000) = 648
*M**_r_* = 304.38	*D*_x_ = 1.243 Mg m^−^^3^
Monoclinic, *C**c*	Mo *K*α radiation, λ = 0.71073 Å
Hall symbol: C -2yc	Cell parameters from 3664 reflections
*a* = 9.966 (2) Å	θ = 3.1–27.5°
*b* = 15.587 (3) Å	µ = 0.08 mm^−^^1^
*c* = 10.477 (2) Å	*T* = 293 K
β = 91.60 (3)°	Block, colourless
*V* = 1626.9 (6) Å^3^	0.38 × 0.32 × 0.27 mm
*Z* = 4	

### Data collection

**Table:**

Rigaku SCXmini CCD diffractometer	1857 independent reflections
Radiation source: fine-focus sealed tube	1361 reflections with *I* > 2σ(*I*)
Graphite monochromator	*R*_int_ = 0.067
ω scans	θ_max_ = 27.5°, θ_min_ = 3.1°
Absorption correction: multi-scan (*CrystalClear*; Rigaku, 2005)	*h* = −12→12
*T*_min_ = 0.967, *T*_max_ = 0.982	*k* = −20→20
8194 measured reflections	*l* = −13→13

### Refinement

**Table:**

Refinement on *F*^2^	Primary atom site location: structure-invariant direct methods
Least-squares matrix: full	Secondary atom site location: difference Fourier map
*R*[*F*^2^ > 2σ(*F*^2^)] = 0.050	Hydrogen site location: inferred from neighbouring sites
*wR*(*F*^2^) = 0.122	H-atom parameters constrained
*S* = 1.02	*w* = 1/[σ^2^(*F*_o_^2^) + (0.0577*P*)^2^] where *P* = (*F*_o_^2^ + 2*F*_c_^2^)/3
1857 reflections	(Δ/σ)_max_ < 0.001
209 parameters	Δρ_max_ = 0.13 e Å^−^^3^
2 restraints	Δρ_min_ = −0.13 e Å^−^^3^

### Special details

**Table:**

Geometry. All e.s.d.'s (except the e.s.d. in the dihedral angle between two l.s. planes) are estimated using the full covariance matrix. The cell e.s.d.'s are taken into account individually in the estimation of e.s.d.'s in distances, angles and torsion angles; correlations between e.s.d.'s in cell parameters are only used when they are defined by crystal symmetry. An approximate (isotropic) treatment of cell e.s.d.'s is used for estimating e.s.d.'s involving l.s. planes.
Refinement. Refinement of *F*^2^ against ALL reflections. The weighted *R*-factor *wR* and goodness of fit *S* are based on *F*^2^, conventional *R*-factors *R* are based on *F*, with *F* set to zero for negative *F*^2^. The threshold expression of *F*^2^ > σ(*F*^2^) is used only for calculating *R*-factors(gt) *etc*. and is not relevant to the choice of reflections for refinement. *R*-factors based on *F*^2^ are statistically about twice as large as those based on *F*, and *R*- factors based on ALL data will be even larger.

### Fractional atomic coordinates and isotropic or equivalent isotropic displacement parameters (Å^2^)

**Table:**

	*x*	*y*	*z*	*U*_iso_*/*U*_eq_	
C1	−0.0405 (4)	0.2124 (2)	0.2764 (4)	0.0609 (9)	
H1A	−0.1100	0.1705	0.2925	0.073*	
H1B	−0.0724	0.2515	0.2102	0.073*	
C2	−0.0017 (5)	0.2605 (3)	0.3966 (4)	0.0862 (13)	
H2A	−0.0749	0.2599	0.4557	0.103*	
H2B	0.0199	0.3196	0.3770	0.103*	
C3	0.1203 (4)	0.2148 (3)	0.4541 (3)	0.0728 (11)	
H3A	0.1014	0.1924	0.5381	0.087*	
H3B	0.1964	0.2534	0.4614	0.087*	
C4	0.1484 (4)	0.1422 (2)	0.3617 (3)	0.0560 (8)	
H4A	0.2443	0.1341	0.3534	0.067*	
H4B	0.1091	0.0890	0.3907	0.067*	
C5	0.0588 (3)	0.09834 (19)	0.1473 (3)	0.0452 (7)	
H5	−0.0119	0.0622	0.1820	0.054*	
C6	0.1826 (3)	0.04266 (18)	0.1305 (3)	0.0447 (7)	
C7	0.1969 (3)	−0.0334 (2)	0.1964 (3)	0.0575 (8)	
H7	0.1296	−0.0480	0.2522	0.069*	
C8	0.3946 (4)	−0.0638 (2)	0.1086 (4)	0.0711 (10)	
H8	0.4690	−0.0994	0.1020	0.085*	
C9	0.3914 (4)	0.0095 (2)	0.0368 (4)	0.0701 (10)	
H9	0.4606	0.0225	−0.0178	0.084*	
C10	0.2826 (3)	0.0636 (2)	0.0475 (4)	0.0587 (9)	
H10	0.2768	0.1137	−0.0007	0.070*	
C11	0.0069 (3)	0.13455 (19)	0.0201 (3)	0.0457 (7)	
C12	0.0554 (3)	0.2112 (2)	−0.0248 (3)	0.0521 (8)	
C13	0.0112 (3)	0.2441 (2)	−0.1444 (3)	0.0596 (9)	
H13	0.0478	0.2949	−0.1744	0.072*	
C14	−0.0833 (4)	0.2028 (2)	−0.2156 (3)	0.0626 (10)	
H14	−0.1114	0.2256	−0.2939	0.075*	
C15	−0.1403 (3)	0.1251 (2)	−0.1726 (3)	0.0528 (8)	
C16	−0.2440 (4)	0.0822 (3)	−0.2422 (3)	0.0635 (10)	
H16	−0.2781	0.1066	−0.3174	0.076*	
C17	−0.2945 (4)	0.0070 (3)	−0.2023 (4)	0.0688 (10)	
H17	−0.3624	−0.0202	−0.2498	0.083*	
C18	−0.2443 (3)	−0.0301 (2)	−0.0888 (3)	0.0614 (9)	
H18	−0.2789	−0.0822	−0.0614	0.074*	
C19	−0.1451 (3)	0.0095 (2)	−0.0180 (3)	0.0526 (8)	
H19	−0.1118	−0.0168	0.0560	0.063*	
C20	−0.0921 (3)	0.08934 (19)	−0.0545 (3)	0.0465 (7)	
N1	0.0857 (3)	0.16953 (15)	0.2391 (2)	0.0476 (6)	
N2	0.2995 (3)	−0.08781 (18)	0.1867 (3)	0.0720 (9)	
O1	0.1471 (2)	0.25894 (14)	0.0428 (2)	0.0616 (6)	
H1	0.1554	0.2398	0.1155	0.092*	

### Atomic displacement parameters (Å^2^)

**Table:**

	*U*^11^	*U*^22^	*U*^33^	*U*^12^	*U*^13^	*U*^23^
C1	0.061 (2)	0.0530 (18)	0.070 (2)	0.0123 (16)	0.0124 (17)	0.0068 (17)
C2	0.105 (4)	0.079 (3)	0.076 (3)	0.026 (3)	0.019 (3)	−0.005 (2)
C3	0.091 (3)	0.072 (2)	0.055 (2)	0.002 (2)	0.007 (2)	−0.0058 (18)
C4	0.065 (2)	0.0566 (19)	0.0461 (17)	0.0057 (16)	−0.0002 (15)	0.0067 (15)
C5	0.0402 (15)	0.0456 (18)	0.0496 (16)	−0.0014 (13)	−0.0030 (13)	0.0079 (13)
C6	0.0420 (16)	0.0414 (15)	0.0502 (16)	−0.0009 (13)	−0.0074 (13)	0.0022 (13)
C7	0.055 (2)	0.0502 (18)	0.067 (2)	0.0037 (15)	−0.0013 (16)	0.0044 (16)
C8	0.057 (2)	0.062 (2)	0.094 (3)	0.0183 (18)	−0.012 (2)	−0.006 (2)
C9	0.051 (2)	0.073 (2)	0.087 (3)	0.0090 (17)	0.0106 (19)	−0.001 (2)
C10	0.0516 (19)	0.0483 (18)	0.076 (2)	0.0023 (15)	0.0050 (18)	0.0067 (17)
C11	0.0413 (16)	0.0467 (18)	0.0490 (17)	0.0039 (13)	−0.0009 (14)	0.0079 (13)
C12	0.0474 (18)	0.0503 (19)	0.059 (2)	0.0009 (14)	0.0017 (16)	0.0061 (15)
C13	0.065 (2)	0.056 (2)	0.058 (2)	0.0037 (17)	0.0039 (18)	0.0202 (16)
C14	0.068 (2)	0.070 (2)	0.0501 (19)	0.0148 (18)	−0.0006 (18)	0.0165 (17)
C15	0.0473 (17)	0.0655 (19)	0.0453 (17)	0.0141 (16)	−0.0021 (14)	0.0004 (15)
C16	0.061 (2)	0.078 (3)	0.0500 (19)	0.0149 (19)	−0.0091 (18)	−0.0046 (18)
C17	0.062 (2)	0.080 (3)	0.063 (2)	0.001 (2)	−0.0129 (18)	−0.016 (2)
C18	0.054 (2)	0.068 (2)	0.062 (2)	−0.0062 (16)	−0.0013 (18)	−0.0053 (18)
C19	0.0503 (18)	0.0544 (18)	0.0529 (18)	0.0018 (14)	−0.0016 (15)	0.0019 (15)
C20	0.0382 (15)	0.0534 (17)	0.0479 (16)	0.0102 (13)	0.0027 (13)	0.0026 (13)
N1	0.0490 (14)	0.0443 (13)	0.0497 (14)	0.0044 (11)	0.0034 (12)	0.0036 (11)
N2	0.067 (2)	0.0563 (19)	0.092 (2)	0.0165 (16)	−0.0065 (19)	0.0065 (17)
O1	0.0666 (15)	0.0538 (13)	0.0643 (14)	−0.0110 (12)	−0.0020 (13)	0.0119 (11)

### Geometric parameters (Å, º)

**Table:**

C1—N1	1.486 (4)	C8—H8	0.9300
C1—C2	1.506 (6)	C9—C10	1.381 (5)
C1—H1A	0.9700	C9—H9	0.9300
C1—H1B	0.9700	C10—H10	0.9300
C2—C3	1.520 (6)	C11—C12	1.376 (4)
C2—H2A	0.9700	C11—C20	1.427 (4)
C2—H2B	0.9700	C12—O1	1.362 (4)
C3—C4	1.520 (5)	C12—C13	1.413 (4)
C3—H3A	0.9700	C13—C14	1.349 (5)
C3—H3B	0.9700	C13—H13	0.9300
C4—N1	1.476 (4)	C14—C15	1.416 (5)
C4—H4A	0.9700	C14—H14	0.9300
C4—H4B	0.9700	C15—C16	1.416 (5)
C5—N1	1.488 (4)	C15—C20	1.428 (4)
C5—C6	1.523 (4)	C16—C17	1.347 (5)
C5—C11	1.524 (4)	C16—H16	0.9300
C5—H5	0.9800	C17—C18	1.402 (5)
C6—C7	1.377 (4)	C17—H17	0.9300
C6—C10	1.380 (4)	C18—C19	1.367 (5)
C7—N2	1.334 (4)	C18—H18	0.9300
C7—H7	0.9300	C19—C20	1.409 (4)
C8—N2	1.324 (5)	C19—H19	0.9300
C8—C9	1.368 (6)	O1—H1	0.8200
			
N1—C1—C2	104.2 (3)	C8—C9—H9	120.9
N1—C1—H1A	110.9	C10—C9—H9	120.9
C2—C1—H1A	110.9	C6—C10—C9	119.3 (3)
N1—C1—H1B	110.9	C6—C10—H10	120.4
C2—C1—H1B	110.9	C9—C10—H10	120.4
H1A—C1—H1B	108.9	C12—C11—C20	119.0 (3)
C1—C2—C3	106.4 (3)	C12—C11—C5	120.4 (3)
C1—C2—H2A	110.5	C20—C11—C5	120.6 (2)
C3—C2—H2A	110.5	O1—C12—C11	122.2 (3)
C1—C2—H2B	110.5	O1—C12—C13	116.8 (3)
C3—C2—H2B	110.5	C11—C12—C13	121.0 (3)
H2A—C2—H2B	108.6	C14—C13—C12	120.8 (3)
C2—C3—C4	104.8 (3)	C14—C13—H13	119.6
C2—C3—H3A	110.8	C12—C13—H13	119.6
C4—C3—H3A	110.8	C13—C14—C15	120.8 (3)
C2—C3—H3B	110.8	C13—C14—H14	119.6
C4—C3—H3B	110.8	C15—C14—H14	119.6
H3A—C3—H3B	108.9	C16—C15—C14	122.3 (3)
N1—C4—C3	105.0 (3)	C16—C15—C20	119.0 (3)
N1—C4—H4A	110.7	C14—C15—C20	118.8 (3)
C3—C4—H4A	110.7	C17—C16—C15	121.7 (3)
N1—C4—H4B	110.7	C17—C16—H16	119.1
C3—C4—H4B	110.7	C15—C16—H16	119.2
H4A—C4—H4B	108.8	C16—C17—C18	119.6 (3)
N1—C5—C6	111.6 (2)	C16—C17—H17	120.2
N1—C5—C11	109.8 (2)	C18—C17—H17	120.2
C6—C5—C11	111.5 (2)	C19—C18—C17	120.6 (4)
N1—C5—H5	107.9	C19—C18—H18	119.7
C6—C5—H5	107.9	C17—C18—H18	119.7
C11—C5—H5	107.9	C18—C19—C20	121.5 (3)
C7—C6—C10	116.9 (3)	C18—C19—H19	119.2
C7—C6—C5	120.4 (3)	C20—C19—H19	119.2
C10—C6—C5	122.6 (3)	C19—C20—C11	123.1 (3)
N2—C7—C6	125.3 (3)	C19—C20—C15	117.4 (3)
N2—C7—H7	117.3	C11—C20—C15	119.5 (3)
C6—C7—H7	117.3	C4—N1—C1	104.0 (2)
N2—C8—C9	124.8 (3)	C4—N1—C5	114.3 (2)
N2—C8—H8	117.6	C1—N1—C5	111.5 (3)
C9—C8—H8	117.6	C8—N2—C7	115.5 (3)
C8—C9—C10	118.1 (4)	C12—O1—H1	109.5

### Hydrogen-bond geometry (Å, º)

**Table:**

*D*—H···*A*	*D*—H	H···*A*	*D*···*A*	*D*—H···*A*
O1—H1···N1	0.82	1.85	2.572 (3)	147
